# 

**Published:** 1884-11-20

**Authors:** 


### EDITORIALS AND MISCELLANEOUS.

### EDITORIAL NOTICES.

SEE advertisements of The Century Company in this number, the Botanical and Zoological Atlases—very valuable and interesting works.

MINERAL EARTH.—See advertisement of this article by National Pharmacy Association in this issue.

DR. JNO. H. LOGAN, of Atlanta, has accepted an appointment to the Chair of Chemistry in Monroe Female College.

DR. S. L. HAMILTON.—Our old and much esteemed medical friend, formerly of Georgia, now of Grandview, Texas, writes us that he is afflicted with cardiac dropsy. We trust that his condition may not prove a serious one.

*Suicide.*—We note the case of suicide by a medical student at the New York University, caused by bad news from his family connections at home.

How foolish, and yet how frequent, these suicides are becoming!

DR. CAMPBELL, of Augusta, now the President of the American Medical Association, has recently been successfully operated upon for cataract. The operation was performed by Dr. Chisolm, of Baltimore.

EXPERT TESTIMONY TO BE REMUNERATED.—Judge White, of Chicago, recently decided in a case where the medical man refused to testify, that he could not be forced to do so without compensation.

WHICH IS IT?—It appears that there are two Medical Colleges, or two Faculties, each acting under one charter, and each claiming to be the Baltimore Medical College. The claimants have gone into the courts.

CHOLERA.—The cholera is reported as subsiding in Europe. In the several places where it has prevailed in France, in Corsica, Algiers, Italy, Spain, the grand total of deaths up to last reports aggregated 19,760, out of about 50,000 cases, or about 30 per cent.

Since penning the above, the disease has broken out afresh in Paris.

DR. J. G. HOPKINS, of Atlanta, has returned to Thomasville, Ga. The Doctor, in a late article in the Atlanta Medical Journal, criticised severely the methods, as a practitioner, of Dr. Wm. A. Hammond, of New York. In the November number of that journal, Dr. Hammond replies, in a communication of equal severity, denying certain of the statements made by Dr. Hopkins, and leaving open some very pointed questions of veracity.

Controversies of a personal character between medical men are to be deprecated.

CLINICS IN THE SOUTHERN MEDICAL COLLEGE.—Many important surgical operations have been performed in the SOUTHERN MEDICAL COLLEGE

the present term, and the clinics in all departments have furnished much practical and useful information to the class.

The Hospital connected with the College is now in good condition, and well equipped in all departments. There is a provision by which medical men having cases, surgical or medical, which they have not the facilities for treating, may send them to the Hospital upon easy and liberal terms. Communications should be addressed to Dr. T. S. Powell, President of Southern Medical College.

### *RECEIPTED.*

1884.—Jno. Gerdine; B. R. Bryant; J. H Jennings; R. J Talbert; E. K. Bozeman to July; J. H. Duggan; M. E. Demaret; M. W. Speer; B. E. Clark; W. P. Anderson; H. S. Bruce; W. M. Peacock; J. T. Kirkpatrick to September; J. H. Reynolds, T. M. Beaty; D. B. Jackson; M. V. Miller; A. H. Sellers; C. M. Gibson.
1886.—J. W. Bennett
1883.—J F. Earnest; G. R. Dozier; B F. Chambliss.
1885.—J. H. W. Young; J. W. Anderson; W. D. Coursey; W. Y. McClure.

